# Horses in the Boer War

**Published:** 1901-07

**Authors:** 


### COMMENCEMENT EXERCISES.

### NEW YORK-AMERICAN VETERINARY COLLEGE.

### NOTES.

### HORSES IN THE BOER WAR.

The following extracts from the debate in the House of Commons on June 6th furnish some curious and interesting information. The House was in Committee of Supply on Army Estimates.

Sir J. B. Maple (Camberwell, Dulwich) asked if it was proposed to bring back all the horses from South Africa. A transport for 400 horses cost £5000 a month. That was equivalent to about £12 per horse, and it seemed to him absurd to bring all these horses back when they would be so much needed in South Africa, and when they could be sold there at a much higher price than could be obtained in this country after the cost of their transport had been taken into account.

Mr. Brodrick said he had been asked if it were the intention to bring back all the horses which had been sent out. He was afraid a great many of the animals had found a permanent home out there ; but the Government did not intend to go to the expense of bringing back ordinary troop horses.

Sir J. B. Maple said that it was important to know whether the War Office was still buying horses from abroad. Last year thousands of perfectly useless horses were bought in Hungary and sent to South Africa, and many of our soldiers lost their lives in consequence of being so badly mounted. He wished to know whether any inquiry was being made into the swindles which had been perpetrated in connection with the purchase of horses at Budapest. It was alleged, too, that the profit which had thus been made had been shared by British officers. He had evidence of the swindles which had been going on from responsible people, and he had submitted it to the Government. If the Government would order an inquiry he would put at their service the special knowledge of his own trainer and of the head of his thoroughbred stud, who knew all that had been going on in Budapest. The authorities in Hungary had been willing to assist the War Office in every way, and they were disgusted at the manner in which the purchases had been made. The horses were the merest rubbish that could have been picked up in the streets ; but they were sold at £25 profit each, and the spoils were divided with certain persons in His Majesty's service. Were horses still being bought from these sources ? This was so important a matter that he thought they ought to ask the Government to grant an inquiry.

Mr. Sinclair (Essex, Romford) said he quite indorsed what had been said. Dealers were engaged to provide a number of horses. They got them from all parts, including Canada and South America, and many of these horses were quite unfit mounts for their men in South Africa. He thought they should have depots throughout the country, and they should induce the farmers to breed a better class of horses. He condemned the middleman system. As to colonial horses, the Australian horses were highly approved, and in Australia they could get horses much cheaper than they paid now. If the colonies were called upon to supply horses it would encourage breeders in those countries. He also urged the utilization of motor cars in certain cases.

Mr. C. Hobhouse said he thought everyone who had heard what the noble lord had said, that full inquiries were being made, would be satisfied with it. The honorable member complained that no allowance was made for the registration of the horses of Transvaal Volunteers intended for transport purposes. Eight millions ($40,000,000) was being spent for the service of remounts. Everyone was agreed that the system of remounts at the beginning of the war was extremely wasteful. According to a letter in *The Times* of June 3d from a correspondent in South Africa, the waste of horses was going on to the same extent; certainly in the same proportion. Horses died, the correspondent said, through being put into requisition before they had time to recover from the effects of the sea voyage. *Much of the waste of horses in the war had been due to the want of skilled veterinary services. If the horses had been better cared for much money would have been saved and our troops would have had greater mobility.* (Italics ours.—ED.)

Lord A. Compton (Beds, Biggleswade) said the lamentable waste of horses had certainly been one of the blots of the campaign in South Africa. His personal experience, as one who had been out of the war, was that the class of horses sent out was not of a good class. He had always believed in English and Irish horses. He bought most of the horses on which his own corps were mounted. He bought them in London at the regulation Government price, and had no difficulty in London in getting plenty of good sound horses. After a bad passage his horses had a few days to recover and again a further opportunity for grass feeding on the march to Bloemfontein. From thence all the horses had hard work and poor feeding, but on arrival at Pretoria the horses of his company were in better condition than those of any of the other troops with whom they were brigaded. Many of the troops had had remounts, and one cavalry regiment had been remounted three times with horses obtained from various parts of the world; and his experience in South Africa confirmed the opinion he had formed after experience in Egypt and India—that if an English horse had a few days to recover after landing he was worth two or three horses from any other country. (Hear, hear.) The subjects of remounts should be included in the general investigation after the conclusion of the war. Our present system of obtaining remounts was essentially bad, and, tested by the war, it had absolutely broken down. He suggested breeding farms, and certainly there should be some system for keeping up the supply of horses for the army.

Lord Stanley said the horses of the company with which his noble friend was connected had not the hard work that fell to the lot of others, though he admitted the corps had arduous outpost duty at Johannesburg. Could the noble lord say the percentage of horses that died?

Lord A. Compton: About 50 per cent.

Lord Stanley said his information was that it was a little higher than that. But by such individual cases the committee should not be led to accept the belief that all the horses sent out were of a bad class. From the commencement of the war to May 31, 172,985 horses were sent out and 80,723 mules, and on May 11th there were 185,000 horses and mules with the forces.

Mr. Pirie said one reason for the extraordinary mortality of the horses was the absolute want of fair play which they received, a great many of them being sent to the front within twenty-four hours of their disembarkation. The orders from the chief of the staff were, however, imperative, and had to be obeyed. Another reason was to be found to some extent in the composition of the irregular corps; for it was not to be expected that engineers, stokers, waiters, and riffraff, men with no knowledge of horses, could be turned into horse masters even in a few weeks. He blamed the Government for their want of foresight in regard to the Remount Department, which in June, 1899, consisted of but one depot in England and one in Ireland, with a total, he believed, of only six officers. The war had not lasted many months before that department had to be greatly enlarged.

Colonel Pilkington (Lancashire, Newton) thought the general conclusion as to the remount system in South Africa was that it had been conducted on a most wasteful principle. The question of remounts was most important. The field army of the future must be to a very great extent a mounted army. Every cavalry regiment ought to have a large number of remounts, so that we could send out without delay not raw animals, but trained, tried, and selected animals. The most perfect scheme for remounts would be one by which in the wild parts of the south of England, of the south of Scotland, and in Ireland horses bought cheaply and selected carefully should be kept ready to remount each of the cavalry regiments at the brigade centre. Our remount system needed revolutionizing. He believed, indeed, that every department in the army wanted revolutionizing. (Opposition cheers.)

Mr. Brodrick said he had no objection to giving the committee generally the prices which the Government had to pay for horses. He thought it was probably to a large extent true that our horses were the best horses. They were also the most expensive horses. The Government paid for cavalry and artillery horses at home on an average about £42; the horses obtained from Canada could be obtained on the spot for about £30; and those obtained in Australia, the United States, and Hungary varied from about £20 to £25. The Government had, of course, to consider the class of horses which could be obtained and the rapidity with which they could be brought together. It was asked why the Government did not take all the horses in the Orange Free State and the Transvaal. Lord Roberts had told him that he had seen very few left in the Orange Free State and the Transvaal, as they had been cleared away by the enemy as they retired. As to Cape Colony, he reminded the committee that without martial law one might buy, but not requisition, horses. When martial law was put into operation horses were requisitioned. As many as 41,000 horses were bought in the month of January in South Africa, and we had been supplying horses at the rate of 10,000 a month for the last six months.

Sir H. Meysey-Thompson (Stafford, Handsworth) said a great deal of the expense and waste in connection with the horses in South Africa had been caused not so much by the want of quality as by the enormous weights, going up to 18, 19, and even 20 stone, which were placed on the backs of the animals. (Hear, hear.) If they wanted to get the best out of the horses, so far as pace and endurance were concerned, they must put light men on their backs. He thought the attention of Parliament and the country ought to be called to the fact that they had been systematically and habitually overweighting their horses, and that they were systematically and designedly restricting the field of recruiting by excluding small, light men from the army. (Hear, hear.)

Captain Norton contended that the action of the Government in connection with the supply of horses for the South African War had been at fault from top to bottom. With regard to the transport of horses, the loss in horseflesh had been extravagant from every point of view. No fewer than fifteen transports left this country at the outset of the war carrying on an average 294 horses each; seventeen transports started out from Australia with a total of 8383 horses, and thirty-two from Buenos Ayres with a total of 25,872. One would have supposed that at least the fifteen transports from this country would have carried a veterinary surgeon. One would have thought that at least that precaution would have been taken when sending valuable horses across the sea, because the horses sent from this country were the most valuable; they averaged £40 per head, when purchased at three and four years old, and when they had been trained were valued at £70 apiece. It might be said that the Government were not aware of the fact that they would require so many veterinary surgeons. But he himself drew attention on the estimates previously to the war to the deplorable state of the Army Veterinary Department, which had only sixty-three executive veterinary officers at the outbreak of the war. With regard to the seventeen ships from Australia and thirty-two from Buenos Ayres, men known as cattle runners were placed on board sixty-four ships, to take care of 38,665 horses. Up to June, 1901, 70,784 reached South Africa from North and South America, Australia, and Hungary. Of the number transported, 3119 died. That was, he admitted, not a large proportion; but of that amount a considerable number would have been saved if veterinary surgeons had been carried by the ships. Of mules there were 70,769 transported, of which 2471 died en route. This was a very large proportion, indeed. To show the loss which had occurred through, as he contended, want of proper supervision, he quoted the case of the M. Battery of the Royal Horse Artillery. That battery was quartered at New Bridge, but it had not its proper complement of horses. The horses were brought out from England without proper inspection, and pink-eye broke out among them, and the whole battery was infected. Again, scratch teams were sent from England which picked up the battery at New Bridge. They were untrained and unknown to the men for service, and that in his opinion was not the proper way to send a battery into action. Then in the case of the ship "Rapidan," on which glanders broke out with most deplorable results, his contention was that the Remount Department ought to be controlled by the Army Veterinary Department, and that all purchases and shipments of horses should be under that department, which would give us a definite department upon which responsibility could be saddled. In the case of all great carrying companies—omnibus and the like—in this country all the horses were bought by the veterinary surgeons, and the results in these cases were fairly good. Another point to which he wished to draw attention was the fact that out of the 250,000 animals which were used in the early stages of the war in South Africa, only 20,000 were purchased in South Africa. In other words, proper advantage was not taken of the opportunity to purchase properly seasoned horses on the spot. The system of station hospitals in India for horses acted admirably, and the Government were obliged to send for some of the staff of those hospitals for service in South Africa, and they were the only hospitals which had done any really satisfactory work. *The loss of so many horses in South Africa was due to the fact that the veterinary surgeons had nobody to give an order to, the farriers and*

*shoeing smiths being under the officers of their own regiments.* The wastage of animals had been stated by the right honorable gentleman to be some 10,000 a month, but this was very greatly under the mark. A few months ago it was about 7500 a week ; between 400,000 and 500,000 animals had been used up during the first seventeen months of the war. The casualties among those animals—he included mules and trek oxen—was 15 per cent., while from mismanagement, starvation, and abandonment on the veldt it was 35 per cent. Most people had read the details which had appeared in the press of the terrible sufferings of the horses which were left to die. It was said that they could not be despatched, because firing in the rear of a force would be a dangerous thing ; but many of these poor beasts might have been despatched with ease with a small poll dagger, such as was used in Spanish bull-fights. England, which ought to be the first country in the world in matters regarding the welfare of dumb animals, was the most backward. *The number of veterinary surgeons required when the army took the field was in Germany one for* 200 *animals; Russia, one for* 120; *France, one for* 313; *Italy, one for* 180. In Austria I have not got the figures for an army on a war footing, but in time of peace they have one veterinary surgeon for 450 animals. We in South Africa had only had one veterinary surgeon to 1200 animals, and the country was surprised that we had these immense losses. In the Transvaal and Natal there were 169 veterinary surgeons, and of these only 38 were army veterinary surgeons. Thus there was only one army veterinary surgeon for between 6000 and 7000 animals. It would be of small value to buy them carefully and to transport them successfully if, when these animals arrived at the seat of war, they were not taken care of. There were many ailments in horses which were of a purely military origin, such as sand colic and acute rheumatism, and other ailments caused by starvation, wrong feeding, overwork, exhaustion, and want of water, and which seldom came within the purview of a civil veterinary surgeon, whose dealings were with horses properly fed and housed. There was one case of an officer's valuable charger which was being led along, and which suddenly lay down and could not be induced to move, which might have been saved had there been proper veterinary assistance. The veterinary surgeons who were sent out upon an emergency—although he did not wish to say anything derogatory to the civil vets.—were the worst class of veterinary surgeons.

The Chairman : Order, order ! The honorable and gallant member is now, I think, dealing with the Veterinary Department, the estimate for which appears on vote 1. He must now confine himself to the question of the horses.

Captain Norton desired respectfully to point out that when he attempted to deal with this matter under vote 1 the chairman had advised him to deal with it under the present vote. His whole point was that out of the whole £36,000,000 which Parliament was called upon to vote last year and this million might have been saved if the suggestion he formerly made had been carried out—*namely, that the Army Veterinary Department should be enlarged and developed.* The success of the entire campaign had been frustrated by the fact that there had not been at disposal a proper supply of properly seasoned and cared-for horses. The delay of seven weeks after the occupation of Bloemfontein was entirely owing to the want of horseflesh, while General French in the ride of his cavalry division from the Modder to Kimberley had 1000 horses unfit for further duty. The cause of all this was insufficient care in purchase and shipment, and also as regards preparation and training, and the want of station hospitals and staff.

Lord Stanley expressed his own belief that this estimate would be sufficient, and that it would cover the expenses up to the close of the war. He hoped that the end of the war was not so far off as many persons believed. He admitted that when he first saw these estimates he felt that this business in Cape Colony would be of long duration. The work was difficult and expensive, but the estimate was taken from a lengthened period, and it would be sufficient for what was required. He admitted that the Remount Department was not up to our requirements, and that it must be reorganized. It would be an advantage to have veterinary surgeons on board ships, but it was impossible to keep up such a large veterinary establishment as to permit each ship to be visited; though some arrangement might be made to provide retainers for men willing to come forward to help the department. As to the loss of horses on the march to Kimberley, he said that the difficulties with regard to water were very exceptional, but it was in the march to overtake Cronje that the loss of horses was so severe. There were seventeen farms or camps where horses were being treated and kept thoroughly well. There were 7268 men employed on these farms, and he hoped by the institution of these establishments to reduce the horse mortality. *The observations of the honorable member with reference to the veterinary surgeons would not be lost sight of.*

Captain Norton said that the complaint was that more than £7,000,000 had been paid during the past year to mount our troops, and that our troops had nevertheless been abominably mounted. *The veterinary department was entirely inadequate, and the civilian veterinary surgeons who had been employed were merely the sweepings of the veterinary profession.—The Veterinary Record.*

The Pennsylvania State Veterinary Association has established a Committee on Animal Husbandry, and President Harger has made the following appointments on the same: Dr. George B. Jobson, Franklin, Chairman; Drs. W. H. Ridge, Trevose; A. W. Radley, Bethlehem; R. S. Huidekoper, Philadelphia; J. C. Michener, Colmar; E. C. Porter, New Castle, and W. H. Fry, Pine Grove Mills, make up the committee. This committee will take up some special work in the interest of matters pertaining to this branch of the Live-stock Association.

The regular meetings of the Ohio State Board of Veterinary Medical Examiners are held on the second Tuesdays of April and July of each year.

The 1901–02 session of the New York American Medical College, Veterinary Department of the New York University, will open on October 1st at 141 West Fifty-fourth Street. The commencement exercises of the school were held at the Metropolitan Opera House, June 6th, at 8 P.M., in connection with the commencement week exercises of the University.

There were eighteen applicants before the Pennsylvania State Board of Veterinary Medical Examiners: fourteen graduates of the Veterinary Department of the University of Pennsylvania; one graduate of McKillip Veterinary College; one of Ontario, and one of New York American Veterinary College.

